# Effect of ^131^I with and without artificial liver support system in patients with Graves’ disease and severe liver dysfunction: A retrospective study

**DOI:** 10.3389/fendo.2022.1034374

**Published:** 2022-10-18

**Authors:** Maohua Rao, Yirui Wang, Jianli Ren, Yue Chen, Chenxi Zheng, Yalan Xiong, Qingbo Yan, Shiying Li, Gengbiao Yuan

**Affiliations:** ^1^ Department of Nuclear Medicine, The Second Affiliated Hospital of Chongqing Medical University, Chongqing, China; ^2^ Chongqing Key Laboratory of Ultrasound Molecular Imaging, The Second Affiliated Hospital of Chongqing Medical University, Chongqing, China; ^3^ Nuclear Medicine and Molecular Imaging Key Laboratory of Sichuan Province, Luzhou, China; ^4^ Key Laboratory of Molecular Biology for Infectious Diseases (Ministry of Education), Institute for Viral Hepatitis, Department of Infectious Diseases, The Second Affiliated Hospital, Chongqing Medical University, Chongqing, China

**Keywords:** thyroid, severe liver dysfunction, artificial liver support system, radioiodine, therapeutics

## Abstract

**Objective:**

Treatment decision-making in Graves’ disease (GD) with severe liver dysfunction (LD) is a clinical challenge. This research was carried out to evaluate the effect of radioiodine (^131^I) with or without an artificial liver support system (ALSS) in GD patients with severe LD.

**Methods:**

In total, 45 patients diagnosed with GD and severe LD were enrolled and allocated to two groups: patients treated with ^131^I alone (n=30) (Group A)and patients by a combination of ^131^I and ALSS (n=15)(Group B). Liver function, thyroid hormone concentrations, therapeutic efficacy, and the cost of treatment were compared between the two groups.

**Results:**

Thyroid hormone concentrations were lower 2 weeks after ^131^I treatment, but no deterioration in liver function was identified. There was no statistically significant difference in the treatment efficacy between the two groups. The hospital stay, total cost, and daily cost were lower in patients treated with ^131^I alone than in those treated with ^131^I and an ALSS (*p*<0.05).

**Conclusion:**

The key point of treating GD patients with severe LD is to control the GD.^131^I is recommended as an effective and safe and should be applied as soon as possible once the diagnosis is clarified; however, when used in combination with an ALSS, there was no substantial improvement in therapeutic efficacy.

## Introduction

Graves’ disease (GD) is an autoimmune thyroid disorder characterized by overproduction and over-release of thyroid hormone (TH) secondary to stimulation of the thyroid-stimulating hormone (TSH) receptor caused by TSH receptor antibody (TRAb) (1). The physiological relationship between the thyroid and the liver is widely recognized and may contribute to the etiology of liver dysfunction (LD) in patients with GD. Previous studies have shown that the mechanism of LD in patients with hyperthyroidism involves elevated oxygen consumption caused by increased metabolic rate; resulting in relative hypoxia in the perivenous zones of liver lobules, which leads to apoptosis and oxidative stress (2). GD may impair hepatic function, resulting in hyperbilirubinemia, liver failure, and even death (3, 4). LD in patients with GD may be caused by the effects of TH excess, drug-related hepatic injury, and the existence of accessory liver disease (5). Moreover, severe LD is a rare but potentially lethal complication of GD, and its treatment is clinically important. Only a few cases of GD with severe LD have been reported to date (6, 7), and there is no clear guidance or expert consensus regarding the management of this clinical condition.

Thyroidectomy, anti-thyroid drugs (ATDs), and radioiodine (^131^I) therapy are the most common treatments for GD. Thyroid resection generally requires treatment with ATDs to reach a euthyroid state prior to surgery. ATDs are not suitable for use in patients who have been diagnosed with GD plus severe liver insufficiency or failure (8–12). For these patients,^131^I therapy is therefore the only option. However, according to the European Association of Nuclear Medicine guidelines (13) and Shen and Liu (3), severe hyperthyroidism with jaundice is a contraindication for ^131^I therapy. Previous study has shown that the TH concentration could increase after ^131^I therapy (14), because of the induction of acute radiation thyroiditis, which is associated with a large release of stored TH into the circulation, resulting in worsening of the symptoms of hyperthyroidism or even a thyroid storm (9, 15).

As a mature method for treating severe LD, artificial liver support system (ALSS) is a device used to help recovering multiple reason-induced LD by *in vitro* mechanical, physical, chemical and biological reaction. The mechanisms of ALSS include molecular adsorbent recirculating system, plasma exchange(PE), or PE in combination with hemofiltration. ALSS can filter and remove kinds of toxins produced by LD, supplementing proteins that are synthesized/metabolized by liver, promoting the hemeostasis, which instantly replaces the basic function of liver and creates an advantageous physiological environment for hepatic repair (16, 17), thus helping with hepatocyte regeneration and liver function recovery (18). A previous study demonstrated that an ALSS improved the safety of ^131^I treatment for patients with GD and LD (19). After the use of the ALSS, the decreases in the mean free triiodothyronine (FT3) and free thyroxine (FT4) levels were 57% and 73% (20), respectively. However, another study showed no significant decreases in the FT3 and FT4 levels (21). Notably, ALSS is high in cost and has certain associated risks (22). Very few studies have focused on how an ALSS affects the recovery of liver function in patients with GD combined with severe LD.

To the best of our knowledge, there has been no comparison of the value of using ^131^I alone or in combination with ALSS for the treatment of patients with GD and severe LD. Therefore, we performed a retrospective study to determine the safety of ^131^I therapy and to compare the efficacy of these two methods of treatment, aiming to provide evidence and guidance for the management of patients with GD and severe LD.

## Materials and methods

### Patients and recruitment criteria

In this retrospective study, 45 patients diagnosed with GD and severe LD were selected from 368 patients who received ^131^I therapy in the Second Affiliated Hospital of Chongqing Medical University from January 2011 to January 2021. The study was approved by the Ethics Committee of the Hospital’s (approval no. 2021E115). The diagnosis of GD was based on typical manifestations including diffuse goiter, thyrotoxicosis, high ^131^I uptake, and positivity for TRAb (23). Severe LD was defined as LD with a prothrombin time activity (PTA) of <60% and/or a total bilirubin (TBil) concentration of >85.5 μmol/L (24, 25). Patients who simultaneously met the diagnostic criteria for both diseases were enrolled. The exclusion criteria was set as: obstructive jaundice, liver cancer, failure of another organ, severe infection, and defective coagulation due to the presence of another disease. The enrolled patients were allocated to two groups: Group A (n=30), in which the patients were treated using ^131^I alone, and Group B (n=15), in which the patients were treated by ^131^I plus ALSS.

### Comprehensive inpatient program

After admission, the patients stopped taking ATDs or other drugs that might cause hepatic failure for quite a long time, including traditional Chinese medicine (TCM) and anti-tuberculosis drugs. Beta-blockers and digoxin were used to control the tachycardia caused by GD since ATDs were withdrawn.

### ^131^I treatment

Patients in both groups accepted ^131^I therapy after commencing a low-iodine diet. Thyroid ultrasonography, ^131^I thyroid scintigraphy, and ^131^I uptake were used to measure the thyroid mass, length and the effective half-life of the iodine. After consultation with more than two nuclear medicine specialists, personalized ^131^I doses were calculated by following formula: therapeutic radioactivity (MBq) = (thyroid mass [g] × ^131^I activity [MBq] per g thyroid tissue)/24-h ^131^I uptake. The patients in Group B underwent ALSS therapy at least once during the ^131^I therapy.

### ALSS

After admission, the Model for End-Stage Liver Disease (MELD) score was compared between the two groups of patients. The MELD score is calculated according to the following formula: MELD = 6.43 + 11.2 × ln [international normalized ratio] + 9.57 × ln (serum creatinine [mg/dL]) + 3.78 × ln (serum bilirubin [mg/dL]) (26).

The patients in Group B underwent ALSS therapy at least once during the ^131^I therapy. For the part, extracorporeal circulation was established by placing a single-needle dual-lumen catheter in the anterior right inguinal vein of each patient,then blood was remained in an anticoagulation state by heparin I.V.The device type was Kawasumi KM-9000, the parameters of ALSS were set as: blood flow velocity was 80-130mL/min; circulating albumin was 80-130ml/min, the dialysate velocity was 500mL/min. Each time of treatment lasted for 4-6 hours. The treatment was repeated every 3-7 days according to the condition of the patients.

### Treatment assessment and follow-up

Laboratory results (including liver function and thyroid function) were obtained 1 to 3 days, 4 to 7 days, 2 weeks,2 and 6 months after treatment, and the hospitalization stay/expenses were collected. The participants were followed for 6 months after discharge. The results of treatment were classified into three levels, including cured, improved, and no response. Cured was defined as both liver function and thyroid function returned to normal or hypothyroidism was present. Improved was defined as liver function and/or thyroid function has improvement. Ne response was defined as neither liver function nor thyroid function has improvement and death. Effective rate equals to the rate of the cured and the rate of the improved.

### Statistical analysis

SPSS 23.0 statistical software (IBM, Inc., Armonk, NY, USA) was used for data analysis. The baseline characteristics of the two groups were analyzed using the independent-samples *t*-test, and the therapeutic efficacy of the two treatments was analyzed using the paired *t*-test. Non-normally distributed data were analyzed using the rank sum test. Odds ratios for the differences between the two groups were calculated using the χ^2^ test, and Fisher’s exact test was used when the expected values were <5. P value <0.05 was considered statistically significant.

## Results

### Patients’ baseline characteristics

As shown in **Table 1**,there were no significant differences in the sex distribution, duration of GD, FT3, FT4, TSH, ALT, AST, TBil, PTA, or ^131^I dose between the two groups. However, there were significant differences in age (*p*=0.026), international normalized ratio (*p*=0.049), and prothrombin time (*p*=0.046). The mean MELD score in Groups A and B was 13.0 ± 4.51 and 15.7 ± 7.38, respectively (*p*=0.07). The patients in Group B tended to have worse liver indices than those in Group A. The three main causes of severe LD were TH excess, drug-related hepatic injury, and viral hepatitis. The detail of the distribution of causes was shown in **Table 1**.

**Table 1:** Baseline characteristics of patients with Graves’ disease and severe liver dysfunction.

	Group A	Group B	P value
Sex(male/female)	17/13	9/6	0.831
Age,year	44.3 ± 13.86	32.7 ± 13.11	0.01
Duration of GD(month)	33.7 ± 67.11	50.7 ± 88.61	0.99
Cause of liver dysfunction,n^a^			0.731
GD induce	16	7	
GD+virus	9	4	
GD+(ATD/other drugs)	(1/4)	(1/3)	
FT3(pmol/L)	18.6 ± 11.93	20.4 ± 9.9	0.367
FT4(pmol/L)	75.3 ± 38.84	82.2 ± 19.9	0.296
TSH(μIU/mL)	0 ± 0.01	0 ± 0	0.798
Alb(g/L)	31.5 ± 5.46	31.2 ± 5.7	0.857
ALT(U/L)	452 ± 482.56	307 ± 432.65	0.413
AST(U/L)	436.9 ± 542.96	339 ± 419.21	0.933
TBil(μmol/L)	295.7 ± 159.82	362.4 ± 141.14	0.178
DB(μmol/L)	215.2 ± 108.15	251.4 ± 119.07	0.312
TBA(μmol/L)	213.7 ± 130.45	269.8 ± 175.57	0.233
INR	1.4 ± 0.37	2 ± 0.94	0.049
PTA(%)	70.9 ± 24.69	55.3 ± 32.54	0.081
PT(s)	16.9 ± 3.64	22 ± 8.49	0.046
MELD	13.0 ± 4.51	15.7 ± 7.38	0.07
Iodine dose(MBq)	262.7 ± 79.18	344.1 ± 138.75	0.077

Data are presented as number of patients or mean ± standard deviation.

INR, international normalized ratio; ALT, alanine aminotransferase; PTA, prothrombin time activity; AST, aspartate aminotransferase; TBil, total bilirubin; FT4, free thyroxine; DB, direct bilirubin; TSH, thyroid-stimulating hormone; TBA, total bile acids; PT, prothrombin time; FT3, free triiodothyronine; MELD, Model for End-Stage Liver Disease; Alb, albumin; GD, Graves’ disease.

a For causes of liver dysfunction.

Reference ranges were as follows: FT3, 2.9–9.1 pmol/L; FT4, 9.1–25.5 pmol/L; TSH, 0.4–5.1 mIU/mL; Alb, 40–55 g/L; TBil, 1.7–17.1 μmol/L; DB, <6.8 μmol/L; TBA, 0–10 μmol/L; INR, 0.7–1.3; PTA, 70%–120%; PT, 9.8–12.1 s; and ALT and AST, both ≤40 U/L.

### Treatment efficacy

The data regarding treatment efficacy at different time points were shown in **Table 2**. At all the time points, no statistically significant difference in the efficacy of the treatments was found between the two groups. One patient died after discharge in Group A, and three in Group B. The death of the patient in Group A, and three Group B. TH replacement was given to six patients who developed hypothyroidism. Six months later, two patients accepted ATD treatment because of recurrence of GD.

**Table 2:** Comparison of therapeutic efficacy of the two treatments.

	Time	Cured	Improved	No response	Effective rate
Group A(n=30)	discharge	–	23	7	76.67%
	Follow-up in two months	–	27	3	90.00%
	Follow-up in six months	8	20	2	93.33%
Group B(n=15)	discharge	–	11	4	73.33%
	Follow-up in two months	1	11	3	80.00%
	Follow-up in six months	3	8	4	73.33%

The efficacy of the two treatments did not significantly differ (χ^2^ = 0.000, 0.216, and 1.947; P=1.000, 0.642, and 0.163, respectively).

### Changes in liver and thyroid indices by ^131^I therapy in Group A

**Figure 1:**
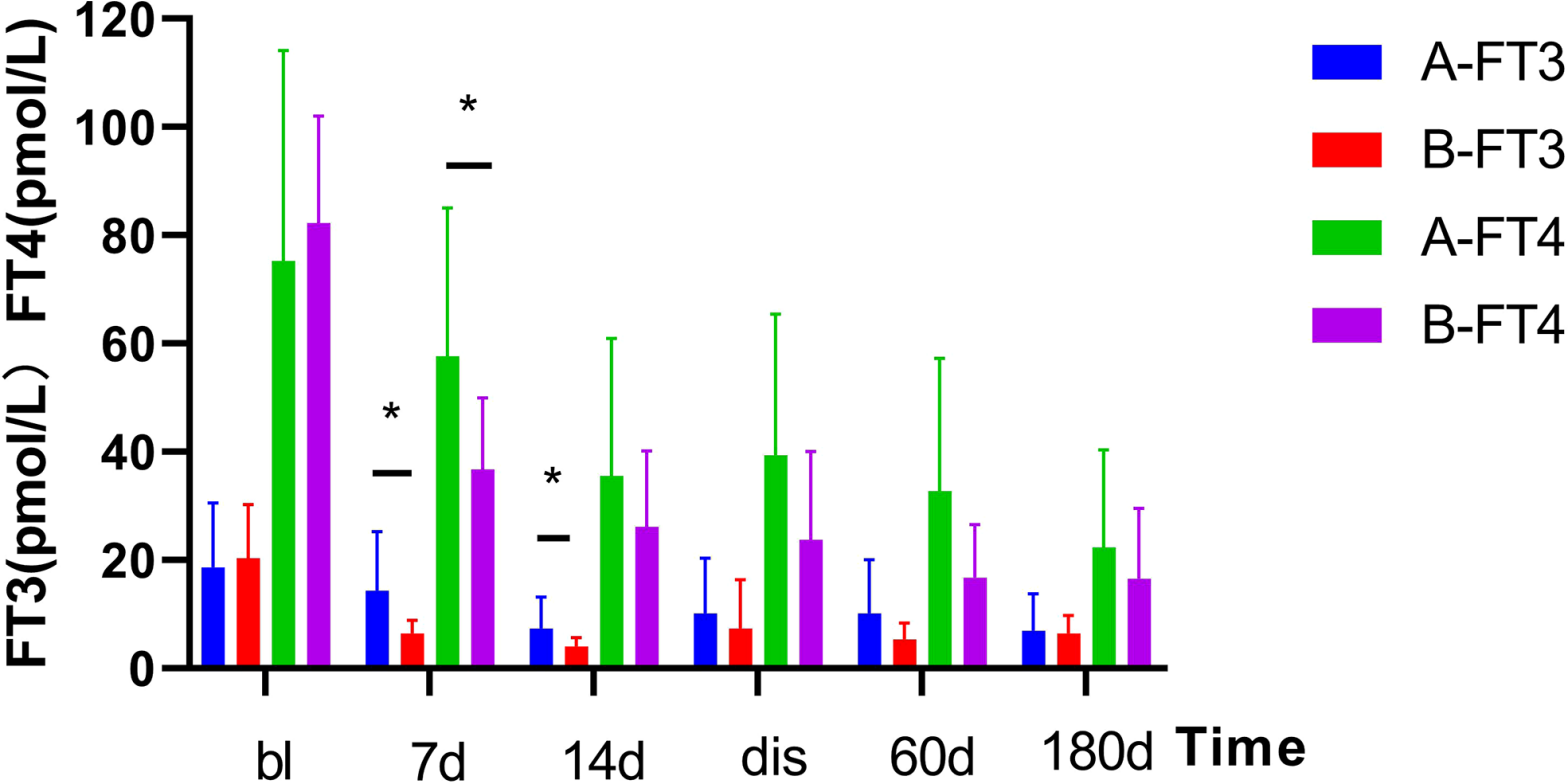
Changes in serum thyroid hormone levels before and after treatment in two Groups. After treatment, the serum thyroid hormone decreased significantly. Results are presented as the mean value ± standard deviation. *Statistically significant difference between the two groups (*p*<0.05). bl, baseline; dis, discharge; FT3, triiodothyronine; FT4, free thyroxine.

**Figure 2:**
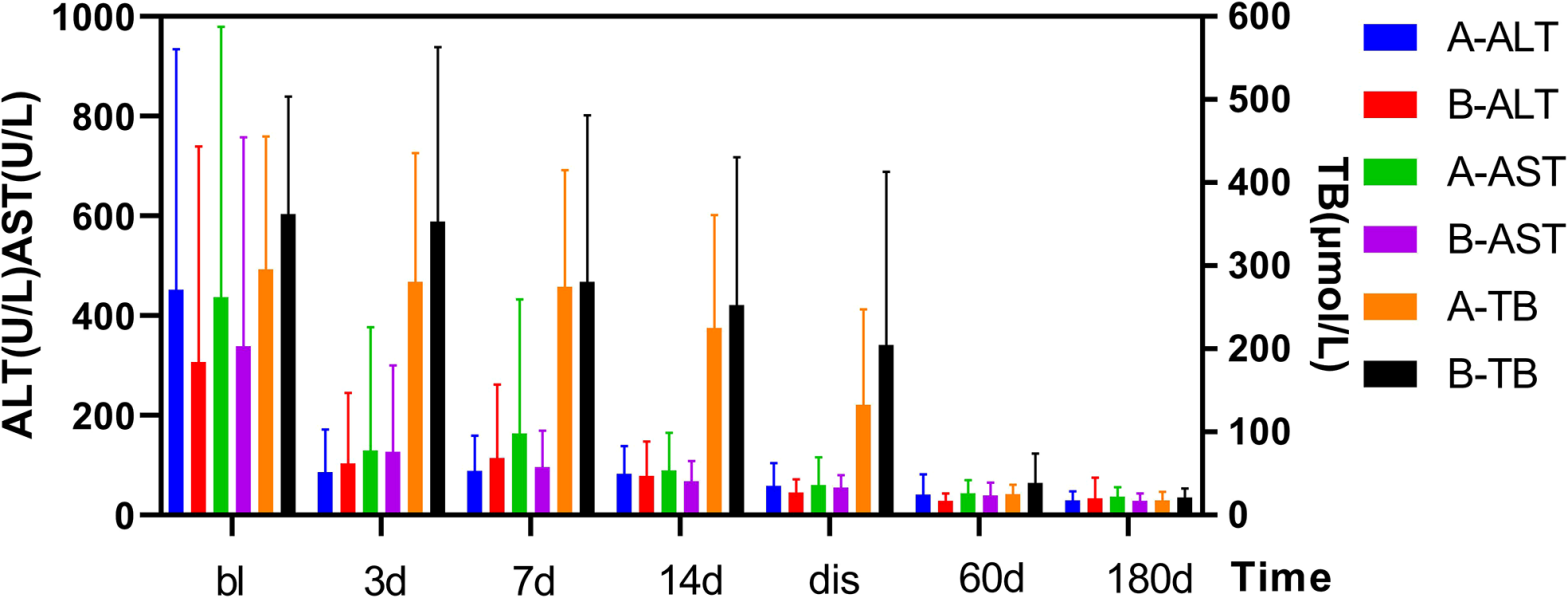
Changes in TBil and ALT/AST levels before and after treatment in two Groups. After treatment, the TBil, and ALT/AST levels decreased significantly. Results are presented as the mean value ± standard deviation. There was not a statistically significant in the TBil, and ALT/AST levels between the two groups. bl, baseline; dis, discharge; TBil, total bilirubin; AST, aspartate aminotransferase; ALT, alanine aminotransferase.

As shown in **Figure 1**. The FT3 and FT4 concentrations significantly decreased after treatment (*p*<0.05):FT3 decreased from baseline(18.6 ± 11.93 pmol/L) to 14.3 ± 10.92 pmol/L 1 week post-treatment and further down to 7.3 ± 5.89 pmol/L at 2 weeks post-treatment, while FT4 decreased from baseline(75.3 ± 38.84 pmol/L) to 57.6 ± 27.47 pmol/L 1 week after treatment, then down to 35.5 ± 25.42 pmol/L 2 weeks after treatment. In **Figure 2**, the liver indices gradually improved after treatment: the ALT (*p*=0.001) and AST (*p*=0.004) activities in Group A significantly decreased within 3 days after ^131^I therapy. The prothrombin time was not significantly lowered until 2 weeks after treatment (*p*=0.014).

### Changes in liver and thyroid indices by combination treatment in Group B

As shown in **Figure 1**. The TH concentration significantly decreased 1 week after the combination treatment (*p*<0.05). FT3 decreased from baseline(20.4 ± 9.9 pmol/L) to 6.4 ± 2.45 pmol/L 1 week after treatment, and 4.14 ± 1.57 pmol/L 2 weeks after treatment, and FT4 decreased from baseline(82.2 ± 19.9 pmol/L) to 36.7 ± 13.26 pmol/L 1 week after treatment and 26.1 ± 14.01 pmol/L 2 weeks after treatment. In **Figure 2**, PTA also significantly changed after 1 week (*p*=0.008). The AST activity significantly decreased 3 days after treatment (*p*=0.037), and the ALT activity (*p*=0.03) and TBil concentration (*p*=0.046) significantly decreased 2 weeks after treatment.

### Comparisons of post-treatment changes in thyroid and liver indices between the two groups

As shown in **Figure 2**, the thyroid and liver indices of both groups significantly improved after treatment. The decrease of FT3 at 1 and 2 weeks after treatment was statistically significant between two groups, and decrease of FT4 was statistically significant at 1 week after treatment. However, no differences in FT3 and FT4 detected at discharge or at follow-up was found to be statistically significant between two groups. In **Figure 2**, there was no significant difference in liver function between the two groups (e.g., TBil, PTA, ALT, AST) after treatment, at discharge, or at follow-up.

### Costs and recovery time

The treatment cost and hospital stay were recorded and compared between the two groups. As shown in **Supplementary Table SA**. The total cost of treatment, mean daily cost, and duration of hospital stay were much lower in Group A than those in Group B, and the difference were statistically significant(p<0.05). The mean length of time required for the patients recovering from severe LD to mild LD was 91.3 ± 56.56 days in Group A and 106.8 ± 69.07 days in Group B (*p*=0.661).

### Changes in TRAb

The changes in TRAb in some patients were shown in **Supplementary Table SB**. After treatment, the TRAb level decreased in both groups, and the decrease in Group B was more obvious, and the difference between two groups was statistically significant.

## Discussion

This study was the first retrospective analysis focusing on the evaluation of ^131^I therapy treating GD with severe LD and the addition of an ALSS. Abnormal liver function is often found in patients newly diagnosed with thyrotoxicosis/hyperthyroidism (prevalence of 15%–76%) (27). A previous study has reported that the incidence rate of severe LD was 6.6% (28). In our research, 12.22% (45 of 368) of GD patients were diagnosed of severe LD. The difference in incidence rate may be explained by admission bias. According to the 2018 edition of the Chinese Guidelines for the Diagnosis and Treatment of Liver Failure, patients who currently have or are at risk of developing liver failure should be managed with ALSS therapy as early as possible (29, 30). In our study, both groups of patients had indications for ALSS therapy, but only patients in Group B received ALSS treatment according to their willingness and their financial status.

The etiology of liver injury in GD patients enrolled in our research varied, and more than one cause were identified. Previously, it has been recommended to recover the functions of both vital organs using multidisciplinary treatment. In this study, the main cause of LD was GD, followed by viral hepatitis and drug-related hepatitis. For a patient with GD-associated LD, the control of LD relies mainly on treating GD. We have observed that LD in 7 patients was caused by TCM, which was possibly due to the unclear chemical composition of TCMs (31).

As shown in **Figures 1**, **2**, after the corresponding treatment, the TH concentration decreased within 2 weeks in both groups, and descended to normal levels during the follow-up for most patients, which was consistent with previous studies that ^131^I therapy is effective for GD patients with LD (32). Previous studies (3, 14) have also reported that the mean TH concentration in patients with GD treated by ^131^I without ATDs pretreatment decreased rapidly. However, results of some studies showed that TH concentration increased after ^131^I treatment (33) or on the day after ALSS (34). There was another study (19) which has reported that the serum concentrations of TBil and FT4 increased 1 week after ^131^I and ALSS. The function of thyroid cells and their TH storage and ATDs all have impacts on serum TH level. We thus hypothesized that the consumption of TH was promoted in GD patients without ATD treatment, whereas the storage of TH was lowered. Thus, after ^131^I treatment, the release of TH were decreased. Conversely, TH level increased rapidly in patients treated by ATDs, This could explain for the different in the TH level after ^131^I treatment as mentioned above. Different from the routine use of ATDs In Western countries, patients in our study did not receive ATDs or stopped receiving ATDs for quite a long time before treatment because of LD.

The key point of treating GD patients with severe LD is the rapid control of the GD, as previously (19). In our study, the liver function was gradually promoted after GD was treated and thyroid function was improved in both groups, and no statistically significant difference in the degree of the improvement in liver function/treatment efficacy/recover time was found between the two groups. At this point, ALSS didn’t show an obvious advantage in the treatment of GD patient with GD patients with LD in our study. However, considering the limitations in sample size and sample bias, further study is required to assess the value of ALSS in the treatment.

The TH concentration in Group B decreased more than that in Group A 1 week after treatment, and the difference was statistically significant. After ALSS treatment in Group B, the level of TRAb decreased by an average of 48% one week after treatment, and a small increase (11%) was detected in 6^th^ months during follow-up. However, the changes of TRAb in Group A were less apparent, showing only a slight decrease (17.4%) during the 6-month follow-up. The data indeed showed a significant ALSS-reduce of TH level, which may be due to the remove of a part of TH by ALSS.

Despite of the uncertainty of ALSS in the improvement of treatment, some of its disadvantages needed to be clarified. First of all, the cost of applying ALSS is significantly higher due to ALSS itself and the prolonged hospital stay. Secondly, ALSS has certain adverse effects, including transfusion reactions, citrate-related nausea and vomiting, hypocalcemia, vasovagal or hypotensive reactions, respiratory distress, catheter dysfunction, bleeding, and tetany or seizure (30, 35). Death is a potential but rare complication. In our study, one patient in Group A was dead due to infectious shock, whereas three patients were dead in Group B due to severe coagulation defect, hepatic encephalopathy and cerebral edema with infection, and heart failure caused by myocardial infarction, respectively. Two of the three patients died in Group B had complications such as hypotension and secondary infection during ALSS treatment. The mortality was 8.89% in this study, where the mortality rate of 2.9% in Group A and 20.0% in Group B.

In conclusion, the key point in the treatment of GD patients with severe LD is the control of GD. ^131^I is recommended as an effective and safe therapy and should be applied as soon as possible once the diagnosis is clarified. The auxiliary treatment by ALSS didn’t show apparent advantage in our study, however, as a preliminary study that requires further confirmation in a larger cohort, we can not negate the function of ALSS in the treatment of GD with severe LD.

## Data availability statement

The original contributions presented in the study are included in the article/**Supplementary Material**. Further inquiries can be directed to the corresponding authors.

## Ethics statement

The studies involving human participants were reviewed and approved by the Ethics Committee of the Second Affiliated Hospital of Chongqing Medical University (approval no. 2021E115). Written informed consent from the participants’ legal guardian/next of kin was not required to participate in this study in accordance with the national legislation and the institutional requirements.

## Author contributions

Conceptualization: MR and SL. Data acquisition and analysis: YW and YW. Writing - original draft: YW and MR. Writing - review and editing: MR, SL, CZ, QY, and YC. Acquistion of funding: JR, MR, and SL. Supervision: GY. All authors contributed to the article and approved the submitted version.

## Funding

This work was supported by a high-level Medical Reserved Personnel Training Project of Chongqing, a Kuanren Talents Program of the Second Affiliated Hospital of Chongqing Medical University (KR2019G001), a Chongqing Science and Health Joint Medical Research Project-Young, a Middle-aged High-level Talent Project (2020GDRC011), an Open Project Program of Nuclear Medicine and Molecular Imaging Key Laboratory of Sichuan Province (HYX20001), and a National Natural Science Foundation of China Youth Fund (81801990). The funding bodies had no involvement in the study design; data collection, analysis, or interpretation; writing of the manuscript; or decision to publish.

## Acknowledgments

We thank Liwen Bianji (Edanz) for edting a draft of this manuscript and YT for her kind assistance in gathering data.

## Conflict of interest

The authors declare that the research was conducted in the absence of any commercial or financial relationships that could be construed as a potential conflict of interest.

## Publisher’s note

All claims expressed in this article are solely those of the authors and do not necessarily represent those of their affiliated organizations, or those of the publisher, the editors and the reviewers. Any product that may be evaluated in this article, or claim that may be made by its manufacturer, is not guaranteed or endorsed by the publisher.

## Abbreviations

GD, Graves’ disease; ALSS, artificial liver support system; LD, liver dysfunction; ATD, anti-thyroid drug; ALT, alanine aminotransferase; PTA, prothrombin time activity; AST, aspartate aminotransferase; TBil, total bilirubin; FT4, free thyroxine; DB, direct bilirubin; TSH, thyroid-stimulating hormone; TBA, total bile acids; PT, prothrombin time; FT3, free triiodothyronine; TH, thyroid hormone; TRAb, thyroid-stimulating hormone receptor antibody; MELD, Model for End-Stage Liver Disease; PE, plasma exchange; TCM, traditional Chinese medicine.
