# Corrigendum: Effect of 131 I with and without artificial liver support system in patients with Graves’ disease and severe liver dysfunction: A retrospective study

**DOI:** 10.3389/fendo.2023.1136544

**Published:** 2023-02-22

**Authors:** Maohua Rao, Yirui Wang, Jianli Ren, Yue Chen, Chenxi Zheng, Yalan Xiong, Qingbo Yan, Shiying Li, Gengbiao Yuan

**Affiliations:** ^1^ Department of Nuclear Medicine, The Second Affiliated Hospital of Chongqing Medical University, Chongqing, China; ^2^ Chongqing Key Laboratory of Ultrasound Molecular Imaging, The Second Affiliated Hospital of Chongqing Medical University, Chongqing, China; ^3^ Nuclear Medicine and Molecular Imaging Key Laboratory of Sichuan Province, Luzhou, China; ^4^ Key Laboratory of Molecular Biology for Infectious Diseases (Ministry of Education), Institute for Viral Hepatitis, Department of Infectious Diseases, The Second Affiliated Hospital, Chongqing Medical University, Chongqing, China

**Keywords:** thyroid, severe liver dysfunction, artificial liver support system, radioiodine, therapeutics

In the published article, there was an error in the author list. The symbol indicating which authors shared co-first authorship was erroneously missed. The corrected author list appears below, using the symbol ^†^ to define which authors share first authorship.

Maohua Rao^1†^, Yirui Wang^1†^, Jianli Ren^2^, Yue Chen^3^, Chenxi Zheng^1^, Yalan Xiong^1^, Qingbo Yan^1^, Shiying Li^4*^and Gengbiao Yuan^1*^

The authors apologize for this error and state that this does not change the scientific conclusions of the article in anyway. The original article has been updated.

