# Supplementary material for: Effect of 131I with and without artificial liver support system in patients with Graves’ disease and severe liver dysfunction: A retrospective study
Source: Front Endocrinol (Lausanne). 2022 Oct 18;13:1034374. doi: 10.3389/fendo.2022.1034374 (PMC9622763; doi:10.3389/fendo.2022.1034374)
Supplement: Supplementary file 1 [file Table_1.pdf]

Supplementary Table A. Costs and recovery time of the two treatments

	Group A	Group B	P value
Total Expenditure (¥)	41868.8±20433.71	114186.7±84903.27	0.001
total days (day)	28.9±13.9	40.2±15	0.033
daily expenses (¥)	1565.9±717.61	2719.7±1222.83	0.008
Recovery time of LD*(day)	91.3±56.56	106.8±69.07	0.661

Data are presented as mean ± standard deviation. LD=liver dysfunction

*Recovery from severe LD to mild LD.
