# Supplementary material for: Effect of 131I with and without artificial liver support system in patients with Graves’ disease and severe liver dysfunction: A retrospective study
Source: Front Endocrinol (Lausanne). 2022 Oct 18;13:1034374. doi: 10.3389/fendo.2022.1034374 (PMC9622763; doi:10.3389/fendo.2022.1034374)
Supplement: Supplementary file 2 [file Table_2.pdf]

Supplementary Table B. Change of TRAb in the two groups

	Pre-treatment	Aft-treatment	6-month-follow-up	*P1*	*P2*
GroupA(n=30)	14.0±12.80	14.9±13.01	12.3±12.50	0.002	0.001
GroupB(n=15)	31.1±10.53	14.2±8.53	15.9±8.59		

Data are presented as mean ± standard deviation.

P1, comparison with values after treatment.

P2, comparison with values at 6-month follow-up.

TRAb = thyroid-stimulating hormone receptor antibody, Aft-treatment = after treatment.
